# In Vitro Assessment of the Cell Metabolic Activity, Cytotoxicity, Cell Attachment, and Inflammatory Reaction of Human Oral Fibroblasts on Polyetheretherketone (PEEK) Implant–Abutment

**DOI:** 10.3390/polym13172995

**Published:** 2021-09-03

**Authors:** Tzu-Yu Peng, Yin-Hwa Shih, Shih-Min Hsia, Tong-Hong Wang, Po-Jung Li, Dan-Jae Lin, Kuo-Ting Sun, Kuo-Chou Chiu, Tzong-Ming Shieh

**Affiliations:** 1 School of Dentistry, College of Dentistry, China Medical University, Taichung 40402, Taiwan; pengtzuyu1014@gmail.com (T.-Y.P.); ll820731@gmail.com (P.-J.L.); djlin@mail.cmu.edu.tw (D.-J.L.); duke111053@hotmail.com (K.-T.S.); 2 School of Dentistry, College of Oral Medicine, Taipei Medical University, Taipei 11031, Taiwan; 3 Department of Healthcare Administration, College of Medical and Health Science, Asia University, Taichung 41354, Taiwan; evashih@asia.edu.tw; 4 School of Nutrition and Health Sciences, Taipei Medical University, Taipei 11031, Taiwan; bryanhsia@tmu.edu.tw; 5 Tissue Bank, Chang Gung Memorial Hospital, Taoyuan 33305, Taiwan; cellww@adm.cgmh.org.tw; 6 Division of Oral Diagnosis and Family Dentistry, Tri-Service General Hospital, National Defense Medical Center, Taipei 11490, Taiwan; scalingdentist@yahoo.com.tw or

**Keywords:** polyaryletherketone, polyetheretherketone, polyetherketoneketone, CAD/CAM, digital dentistry, implant–abutment, cell metabolic activity, cytotoxicity, human oral fibroblast, pro-inflammatory cytokines

## Abstract

The purpose of this research is to compare the cytotoxicity of polyetheretherketone (PEEK) and polyetherketoneketone (PEKK) with conventional dental implant–abutment materials, namely titanium alloy (Ti-6Al-4V) and yttria-stabilized tetragonal zirconia polycrystal (Y-TZP), to evaluate the cell metabolic activity, cytotoxicity, and inflammation potential of human oral fibroblasts (HOF) on these materials. Disk-shaped specimens were designed and prepared via a dental computer-aided manufacturing technology system. Surface topography, roughness, and free energy were investigated by atomic force microscope and contact angle analyzer; cell metabolic activity and cytotoxicity by MTT assay; and morphological changes by scanning electron microscopy (SEM). The effect of pro-inflammatory gene expression was evaluated by RT-qPCR. The obtained data were analyzed with one-way analysis of variance and post-hoc Tukey’s honest significant difference tests. PEEK and PEKK exhibited higher submicron surface roughness (0.04 μm) and hydrophobicity (>80°) than the control. Although the cell activity of PEEK was lower than that of Ti-6Al-4V and Y-TZP for the first 24 h (*p* < 0.05), after 48 h there was no difference (*p* > 0.05). According to the cell cytotoxicity and the pro-inflammatory cytokine gene expression assays, there was no difference between the materials (*p* > 0.05). SEM observations indicated that HOF adhered poorly to PEKK but properly to Ti-6Al-4V, Y-TZP, and PEEK. PEEK and PEKK show comparable epithelial biological responses to Ti-6Al-4V and Y-TZP as implant–abutment materials. Between the two polymeric materials, the PEEK surface, where the HOF showed better cell metabolic activity and cytotoxicity, was a more promising implant–abutment material.

## 1. Introduction

Polyaryletherketone (PAEK) is a semi-crystalline high-performance thermoplastic polymer whose molecular backbone is linked by phenylene rings (aryl), oxygen bridges (R-O-R), and carbonyl groups (R-CO-R) [1]. The phenylene rings are unreactive, the ether group (R-O-R) provides flexibility, and the ketone group (R-CO-R) rigidity; therefore, the combination of the three functional groups creates PAEK with excellent resistance to chemical attack, good toughness, and high strength, combined with heat resistance and good processability [2,3,4]. The PAEK family has many members according to the different sequences and ratios of aryl, R-CO-R, and R-O-R in the chemical structure [5]. Polyetheretherketone (PEEK) has recently attracted considerable attention due to its esthetic properties and has been successfully introduced into the dental field [1,4]. PEEK is very useful in digital dentistry [6] and in general when immediate loading [7] or preimplant surgery procedures [7,8] are requested; moreover, PEEK displays good shock absorption and fatigue and abrasion resistance and is highly stable in the oral cavity without undergoing physicochemical changes [2,4,9]. Additionally, PEEK overcomes the problems caused by the brittle properties of ceramic materials and artificial techniques, such as staining, which can adjust the final color, thereby conquering the unpleasing appearance of alloy materials [10]. PEEK restorations can be easily manufactured via computer-aided design and computer-aided manufacturing technology (CAD/CAM) [4,9]; because of the rapid development of digital dentistry, the digital files are easily preserved, so when failure occurs, the PEEK restorations can be reproduced immediately [3,11]. PEEK has a relatively close elasticity modulus (approx. 4 GPa) to human bone (approx. 14 GPa), exhibits similar tensile properties (approx. 80 MPa) to natural enamel (approx. 68 MPa) and dentine (approx. 104 MPa) compared to titanium alloy (approx. 110 GPa, 1200 MPa) and zirconia (approx. 210 GPa, 550 MPa) [1,2], and has the capability of being combined with other materials [4,12,13]. The shock-absorbing effect provided by PEEK might moderately absorb and disperse the impact under occlusion and chewing force, achieving a stress-dispersion mode like natural teeth [1,3,4,9]. Titanium osteosynthesis materials may cause complications over time and may need to be removed [14]; however, owing to the natural properties of PEEK, it can be used as a bone substitute in tissue engineering [15]. Thus, when applying PEEK as an alternative implant–abutment material to replace traditional materials, titanium alloy, or recent widely used materials, zirconium dioxide can be used as an implant–abutment material to reduce the risk of failure, eliminate concerns about metal allergies, and improve patient safety and esthetic satisfaction, thereby improving quality of life [10,16].

When dental materials are used as an implant–abutment, bacteria or microorganisms must not be allowed to adhere on the surface, because this results in biofilm formation around the implant and abutment, which will later cause periodontitis and peri-implant mucositis [17,18]. This undesirable response not only causes a psychological burden for the patient, but it also increases the possibility of early implant failure [19,20,21]. After the insertion of implant–abutments, the surrounding fibroblasts, and keratinocytes trigger regenerative procedures to generate the underlying collagen matrix and cover the epithelial keratinocyte layer, respectively, around the implant–abutments through cell migration from a soft tissue barrier [22,23]. This barrier enables the soft tissue around implant–abutment to serve as a protective seal with the bones around the implant–abutments and below [24], which not only protects the implant–abutment connection from peri-implant mucositis caused by the invasion of exogenous and noxious bacteria but also reduces the possibility of early implant failure caused by bone loss [22,25], thereby extending the lifespan of dental implant–abutments. To sum up the above, the critical factors for the application of dental materials to dental implant–abutments is that the materials are first not prone to biofilm formation, and second are apt to enable cell adhesion.

Based on the various merits discussed above, as well as its comfortability and stability in the oral cavity, PEEK is an alternative dental material for implant–abutments that is worthy of discussion [1,4,13]. In a previous study, the authors discussed the biofilm formation characteristics of PEEK and some oral bacteria, confirming that PEEK is not prone to biofilm formation [26]; nevertheless, the biological response of oral cells and fibroblasts to PEEK remains to be verified. The present experiment discusses the possibility of applying PEEK materials in dental implantology. The null hypothesis of the present study is that PEEK shows cell metabolic activity, cell adhesion, and pro-inflammatory cytokine responses to human oral fibroblast (HOF) comparable to traditional implant–abutment materials.

## 2. Materials and Methods

### 2.1. Specimen Preparation

Three categories (metallic, ceramic, and polymeric) of four different dental materials (Ti-6Al-4V, Y-TZP, PEEK, and PEKK) were used in the present study. The detailed material information is presented in Table 1. The testing specimens were designed as disk-shaped specimens with a diameter of 10.0 mm and a thickness of 2.5 mm by SolidWorks (2013 version; Dassault Systèmes SolidWorks, Waltham, MA, USA), and fabricated using a dental CAD/CAM milling machine (Zirkonzahn M1; Zirkonzahn GmbH, Gais, Italy). All the specimens were wet-ground with silicon carbide abrasive paper (W/D Sheet; Kovax Corp., Tokyo, Japan) of grades #600, #1000, and #1500. Subsequently, all the specimens were washed with de-ionized water in an ultrasonic cleaner (Soner 220H; Rocker Scientific Co., Ltd., New Taipei City, Taiwan) and air dried.

### 2.2. Surface Characterization

#### 2.2.1. Surface Roughness, Topography, and Morphology

The atomic force microscope (AFM) (Bruker Dimension Icon VT-1000; Santa Barbara, CA, USA) was used and the silicon probe in tapping mode was selected to obtain the surface topographies and submicron surface roughness (Ra) of a flat, 5 × 5 μm^2^-sized portions of the surface. Three specimens were evaluated in each specific material to determine the average, which was then used to evaluate the final Ra value of each material. The surface morphology was observed using a thermal-field emission scanning electron microscope (FE-SEM) (JEOL JSM-7800F Prime; JEOL Ltd., Tokyo, Japan). The specimens were primarily gilded with platinum under 10 mA/25 s (JEOL JEC-3000FC Auto Fine Coater JEOL Ltd., Tokyo, Japan), and the images were recorded at × 1k magnification.

#### 2.2.2. Hydrophilicity and Surface Free Energy (SFE)

The hydrophilicity of the test specimens was assessed at room temperature using a contact angle analyzer (FTA-125; First Ten Angstroms, Inc., Newark, CA, USA). An approximately 10 μL droplet of de-ionized water was vertically extruded from a 31G needle onto the testing specimens and a continuously recording charge-coupled device (CCD) was triggered to record it. Each reported contact angle was the mean of ten independent measurements for each material (n = 5). Since the present study only used deionized water as a testing liquid, the surface free energy (SFE) was calculated by the Girifalco–Good–Fowkes–Young (GGFY) model [27] with the software program (FTA32; First Ten Angstroms, Inc., Newark, CA, USA) set to the contact angle data obtained described above.

### 2.3. Biological Evaluation

#### 2.3.1. Cell Cultures

The tissue of primary human oral fibroblast (HOF) culture was donated by patients who had signed the informed consent form in the dental clinic of the China Medical University Hospital. The HOF cultures were seeded and cultured in Dulbecco’s modified Eagle medium (DMEM; Caisson Laboratories, North Logan, UT, USA) containing 10% fetal bovine serum (FBS) and 1% antibiotic/antimycotic at 37 °C in an atmosphere of 5% CO_2_ in 100 mm culture dishes [28].

#### 2.3.2. Cell Metabolic Activity

All the specimens were autoclaved (121 °C, 1.2 kg/cm^2^, 15 min) before the cellular experiments. The testing materials were transferred to a 24-well plate and HOF cultures were seeded on top of each specimen at a density of 3 × 10^6^ cells/well. The HOF cultures were incubated in direct contact with the materials for 24, 48, and 72 h. To quantify the potential cell metabolic activity, 0.5 mL fresh prepared 1 mg/mL [3-(4,5-dimethylthiazol-2-yl)-2,5-diphenyltetrazolium bromide] (MTT) reagent (M6494; Thermo Fisher Scientific Inc., Waltham, MA, USA) was added to each well and incubated for 4 h at 37 °C. The supernatant was removed, and the cells were washed with PBS. The blue formazan product was solubilized with 1 mL of dimethylsulfoxide (DMSO; Mediatech. Inc., Manassas, VA, USA). After solubilization, 50 μL of liquid from each well was transferred to a 96-well plate. The absorbance was determined at 590 nm (OD590) using an ELISA reader (VersaMax; Molecular Device, San Jose, CA, USA) [29]. The experiments were replicated three times, and the results were calculated as the averaged absorbance of all replicates. The values were then normalized against the Ti-6Al-4V 24 h group.

#### 2.3.3. Cell Adhesion and Morphology

Specimens from each testing group (Ti-6Al-4V, Y-TZP, PEEK, and PEKK) were placed in 60-mm culture dishes and seeded with HOF for 48 h following the protocols described above. All the testing specimens were then cleaned with PBS, followed by 4% formaldehyde fixation and alcohol dehydration, before being dried to the critical point. HOF attached to each testing specimen was observed with FE-SEM (JEOL JSM-7800F Prime; JEOL Ltd., Tokyo, Japan). The images were taken at a magnification of ×2k.

#### 2.3.4. Cell Cytotoxicity

The specimens of each testing material (Ti-6Al-4V, Y-TZP, PEEK, and PEKK) were immersed in DMEM supplemented with antibiotics for 72 h at 37 °C to prepare the extracts for the cell cytotoxicity assay. HOFs were seeded in a 96-well plate (1 ×10^6^ cells/well). Subsequently, the old medium was replaced by extracts. DMEM was used only as a control after treatment for 48 h following the MTT protocols described above to quantify the cell cytotoxicity. The experimental result is the average absorbance of three replications, and all values were normalized with DMEM.

#### 2.3.5. Pro-Inflammatory Cytokine Gene Expression

The HOF cultures were inoculated with extracts of each testing material for 48 h in 60-mm culture dishes. Lipopolysaccharide (LPS) treatment was regarded as a positive control. The cells were then harvested for the reverse transcription-quantitative polymerase chain reaction (RT-qPCR) analysis. Total RNA from the HOF was extracted using TRI reagent (Molecular Research Center, Inc., Cincinnati, OH, USA). Reverse transcription (RT) of the total RNA was performed using a random primer, and the cDNA was used as the PCR template. The expression of pro-inflammatory genes *interleukin-1β* (*IL-1β*), *interleukin-6* (*IL-6*), and *tumor necrosis factor-α*, (*TNF-α*) was normalized with *glyceraldehyde 3-phosphate dehydrogenase* (*GAPDH*) expression. All tests were conducted in triplicate. The data analysis followed the method described by Chiu et al. [30]. The primer sequences of each gene are listed in Table 2.

### 2.4. Statistical Analysis

All data shown in figures and tables were depicted as the mean ± standard deviation (SD). The Shapiro–Wilk test and Levene’s test confirmed that all the data were normally distributed and homogeneous; therefore, parametric tests were used in this study. The experiments replicated three times for each specific assay. The comparisons of the data were conducted via one-way analysis of variance (ANOVA) and the multiple comparisons of different testing groups (Ti-6Al-4V, Y-TZP, PEEK, and PEKK) were analyzed using post-hoc Tukey’s honest significant difference (HSD) test. All statistical analyses were performed using SPSS statistical software (version 24; IBM Corp., Armonk, NY, USA).

## 3. Results

### 3.1. Surface Characterization

**Figure 1:**
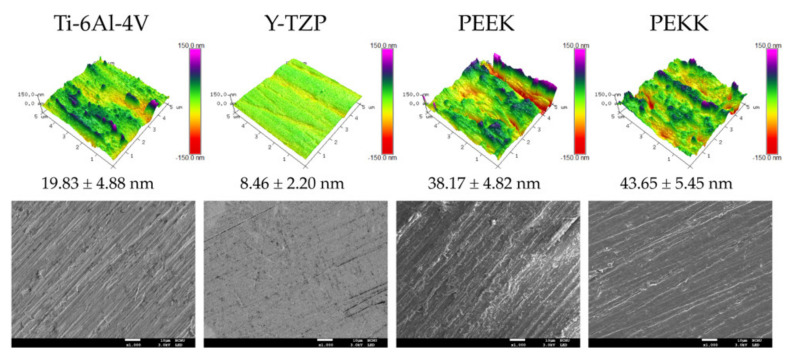
First row: 5 × 5 μm^2^ AFM images and the submicron surface roughness (Ra) taken from the flattened surface of each testing material. The surface topography is presented as different color ranges, from +150 nm (purple) to −150 nm (red), and the Ra values are shown under each image (mean ± standard deviation (SD), n = 3). Second row: FE-SEM microphotographs of surface morphologies. The scale bars are 10 μm (original magnification ×1000).

Figure 1 illustrates 5 × 5 μm^2^ AFM images and the submicron surface roughness (Ra) for each testing material. PEEK (Ra = 0.04 μm) and PEKK (Ra = 0.04 μm) presented higher submicron roughness compared to the Ti-6Al-4V (Ra = 0.02 μm) and Y-TZP (Ra = 0.01 μm) groups. The FE-SEM microphotographs of surface morphologies are shown in the second row of Figure 1. After treatment with silicon carbide abrasive paper, the surface of the Y-TZP group was relatively smooth, with only slight unevenness. In contrast, the PEEK and PEKK samples showed distinct scratches and roughness.

Table 3 shows the contact angle (hydrophilicity) values and surface free energy (SFE) values for all the testing materials. For hydrophilicity, the results indicated that PEEK and PEKK had significantly higher contact angles (*p* < 0.05) than that of the controlled Ti-6Al-4V and Y-TZP. In comparison, PEEK (80.91°) and PEKK (84.03°) were not significantly different (*p* = 0.07). The lowest contact angles were observed for Ti-6Al-4V (65.83°) followed by Y-TZP (76.92°) samples. For SFE results, the two polymer materials (PEEK and PEKK) had significantly lower values (*p* < 0.05) than those of Ti-6Al-4V and Y-TZP. However, there was no difference between PEEK and PEKK (*p* = 0.34). The lowest SFE values were observed for PEKK samples (22.19 mN/m), followed by PEEK (24.41 mN/m) and Y-TZP (27.38 mN/m), whereas Ti-6Al-4V samples (36.25 mN/m) showed the highest values of SFE.

### 3.2. Cell Metabolic Activity

**Figure 2:**
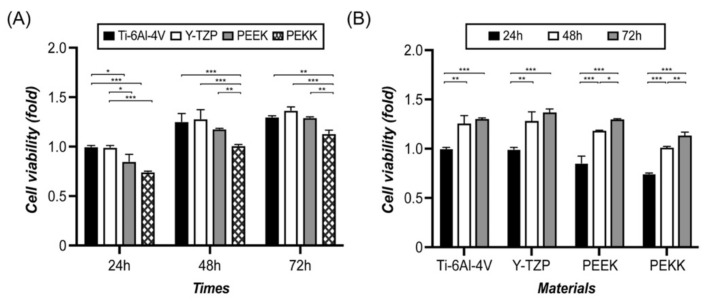
Cell metabolic activity of HOF in direct contact with the surface of the abutment material (Ti-6Al-4V, Y-TZP, PEEK, or PEKK) for 24 h, 48 h, and 72 h, assessed through MTT assay. Significant difference is based on a one-way ANOVA with Tukey’s multiple comparisons test to compare (**A**) the differences in effect between the materials and (**B**) the effect within the same material over specific time periods. (* *p* < 0.05, ** *p* < 0.01, *** *p* < 0.001).

The cell metabolic activity of HOF in direct contact with the testing material (Ti-6Al-4V, Y-TZP, PEEK, or PEKK) is shown in Figure 2. Based on the statistical analysis of the results of the MTT assay, under the same “culture time” (Figure 2A), the PEKK group was significantly lower than the Ti-6Al-4V and Y-TZP (*p* < 0.001) control groups. However, the PEEK group and the two control groups were only significantly different for 24 h, but not after 48 h or 72 h. Regarding the same material (Figure 2B), culture time significantly affected cell metabolic activity (*p* < 0.05), especially for the two polymer materials (PEEK and PEKK); the results show a significantly positive increase between the three culture time periods, e.g., 24 h to 48 h (*p* < 0.001), 24 h to 72 h (*p* < 0.01), and 48 h to 72 h (*p* < 0.05). These results indicate that HOF attached to the surface of PEKK, and that PEEK was weaker than Ti-6Al-4V or Y-TZP.

### 3.3. Cell Adhesion and Morphology

**Figure 3:**
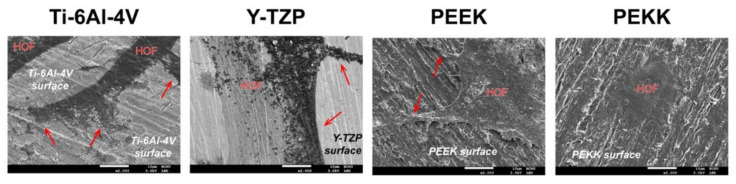
FE-SEM microphotographs obtained after 48 h, when HOF had adhered and proliferated on the surface of the abutment materials (Ti-6Al-4V, Y-TZP, PEEK, and PEKK). The scale bar is 10 μm (original magnification ×2000). The arrows indicate the cell pseudopodia.

The cell adhesion mode and morphology of the HOF cells on different materials was observed via FE-SEM (Figure 3). According to Figure 3, it was apparent that the spreading of HOF cells was poor in the PEKK group. However, the flat, elongated (spindle-shaped) cells typical of HOF could be observed on the other three materials (Ti-6Al-4V, Y-TZP, and PEEK). Under more detailed observation, cell attachment morphology of HOF on the Ti-6Al-4V and Y-TZP surfaces was evenly distributed and tightly attached to the material’s surface, indicating good adhesion. The pseudopodia structures of HOF cells, used to grip a surface, were most apparent on PEEK materials, revealing their better affinity for that material. However, the HOF cells adhered poorly to the PEKK material, appearing agglomerated, and pseudopodia structures could not be observed.

### 3.4. Cell Cytotoxicity

**Figure 4:**
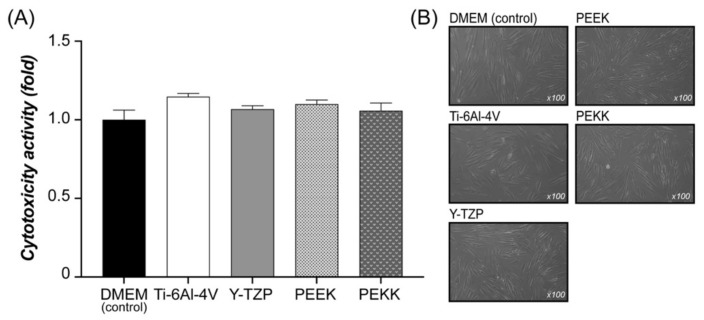
(**A**) Assessment of cytotoxicity activity by indirect exposure of HOF to extracts of the abutment materials (Ti-6Al-4V, Y-TZP, PEEK, and PEKK) through MTT assay. (**B**) The optical microscope image of HOF morphology in the extract of the abutment materials (original magnification ×100).

For cell cytotoxicity testing, the HOF cultures were exposed to extracts of Ti-6Al-4V, Y-TZP, PEEK, and PEKK (Figure 4A). Based on the results of a one-way ANOVA with Tukey’s multiple comparisons test of the MTT assay, the stimulation of cytotoxicity activity observable in HOFs exposed to different extracts was not statistically significantly different (*p* > 0.05) to control cultures (DMEM). Nonsignificant differences (*p* > 0.05) were also found when comparing different extracts. Observing the optical microscope image (Figure 4B), the similarity in the HOF morphology and cellular density can be seen for each specific extract and control.

### 3.5. Pro-Inflammatory Cytokine Gene Expression

**Figure 5:**
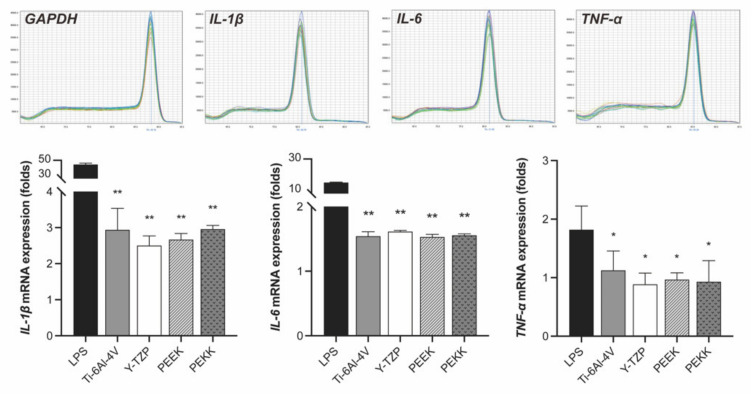
Gene expression of pro-inflammatory cytokines (*IL-1β*, *IL-6*, and *TNF-α*) in HOF after 24 h of cultivation in testing extracts (Ti-6Al-4V, Y-TZP, PEEK, and PEKK). One-way ANOVA with a post-hoc Tukey HSD test was conducted for multiple comparisons with the LPS group. (* *p* < 0.05, ** *p* < 0.01).

Figure 5 shows the gene expression of three pro-inflammatory cytokines, *IL-1β*, *IL-6*, and *TNF-α*, in HOF after 48 h of cultivation in each extract (Ti-6Al-4V, Y-TZP, PEEK, or PEKK). The mRNA expression of *IL-1β* cytokines (*p* < 0.01), *IL-6* (*p* < 0.01), and *TNF-α* (*p* < 0.05) was statistically significantly different between the various materials and the positive control (*LPS*). However, comparison of the materials did not show that any of them statistically significantly promote pro-inflammatory gene expressions (*p* > 0.05).

## 4. Discussion

Polyetheretherketone (PEEK) and polyetherketoneketone (PEKK) are the two major members of the PAEK family used in the dental biomedical sciences [1,4,5]. The present study confirmed that under the same culture conditions for human oral fibroblast (HOF), PEEK displayed comparable cell metabolic activity, cell adhesion, and pro-inflammatory responses to traditional implant–abutment materials (i.e., Ti-6Al-4V and Y-TZP). Therefore, the null hypothesis is supported, and it is preliminarily confirmed that PAEK, especially PEEK, is a novel and potentially useful material for dental implant–abutments.

Y-TZP has a high hardness of 1200 Hv [31], and a significantly lower submicron surface roughness than other experimental groups under the same surface treatment conditions (*p* > 0.01). From the AFM and SEM images, it can be seen that the Y-TZP surface was almost smooth (Figure 1). PEEK and PEKK have a low hardness of approximately 26.1 to 28.5 Hv [32]. PEEK materials are medium viscosity materials, and, accordingly, observation of the SEM images shows that PEEK and PEKK had a filamentary morphology (Figure 1). The hardness of Ti-6Al-4V (300 Hv [33]) is lower than that of Y-TZP, but without viscosity, so although the submicron surface roughness was also minor, the surface morphology showed a uniform and noticeably line-like texture (Figure 1). Bone remodeling around dental implants is regulated according to the load applied on the implant fixture. Previous experiments that discussed the cytotoxicity of PEEK for MG-63 concluded that the cell metabolic activity of MG-63 on PEEK was similar to Ti-6Al-4V and Y-TZP [26]. Titanium dental implants are currently viewed as the gold standard in dental implantology. As with titanium alloy, zirconia and PAEK are bioinert. When used as dental implant components, their osseointegration ability is no different. However, the focal point of the current study was the application of PEEK as an implant–abutment. Occlusal loads distributed on implants are important for the long-term success of implant-supported prostheses [34]. Y-TZP has a high fracture toughness, from 5 to 10 MPa m^1/2^, and a flexural strength of 900–1400 MPa [35]. These excellent mechanical properties have always been a double-edged sword for Y-TZP when used in dental implant restorations (e.g., as implant fixtures, abutments, or crowns). When occlusal force is applied to Y-TZP implant restorations, the stress is directly transferred [36]; this can cause not only implant failure, but also damage to the alveolar bone [37]. Besides this, when Y-TZP is selected as the crown material for implant restorations, if zirconia is not used, chipping and fracturing occur easily [38]. PEEK and PEKK are flexible materials with a similar elasticity modulus (3–8 GPa) to human hard tissues and bones [1,9]. Using PEEK as an implant–abutment can reduce stress concentration on the surface and exert a shock-relieving effect [3,4]. Scholars have analyzed the stress distribution of PEEK as an implant material through finite element analysis [36,39]. Their experimental results confirm that PEEK can adequately distribute force to the alveolar bone, which reduces the early failure rate of implants and harm to the alveolar bone.

The basic requirement for fibroblasts to survive on a material is cell adhesion. Only then can cellular physiological phenomena, such as cell diffusion, migration, proliferation, and differentiation, occur. This can help with collagen secretion, wound healing, and tissue guided regeneration. The roughness, mechanical properties, wettability, and surface energy of substrate surface affect cell adhesion [40]. In general, PAEK materials have higher hydrophobicity and lower surface energy than metallic or ceramic materials due to the presence of fewer polar functional groups on PAEK surfaces, and the results from the current study were consistent with this [41]. Discussing the two PAEK materials, we found that PEEK, with more ether groups, exhibited a lower contact angle and higher surface energy than PEKK. Ether molecules do not have a hydroxyl group (-OH), and hydrogen bonds could not bond between ether molecules; however, there are lots of non-bonding pairs on the oxygen atom of the ether molecules, which can form hydrogen bonds with -OH or N-H. Therefore, PEEK has relatively high polarity. Whether cells attach to the material’s surface depends on the focal adhesion sites. PEEK, with higher polarity than PEKK, makes it easy for cells to adhere to specific cell receptors (i.e., integrins) on the cell membrane through attachment proteins (i.e., fibronectin, collagen) and thus adhere to the surface [42]. The result of the MTT assay of HOF directly cultured on the material’s surface (Figure 2) confirms that PEEK had better cell attachment and adhesion than PEKK at any time point (24 h, 48 h, 72 h). The adhesion and morphology of HOF on its surface, according to SEM observation (Figure 3), confirm that PEEK had better biological behavior than PEKK.

The survival rate of dental implants depends on whether the implant–abutment will cause inflammation after insertion. The current results showed no significant cytotoxicity (Figure 4), nor evident pro-inflammation genes expression (Figure 5), and so confirm that PEKK and PEEK are materials with similar cytotoxicity to Ti-6Al-4V and Y-TZP. Administration of antibiotics treats bactericidal diseases with good effect, but antibiotics have the problem of drug resistance and the risk of allergic reaction. Polizzi et al. [43] indicated that chlorhexidine allows good control of the clinical indices. Peng et al. [26] confirmed that the risk of bacterial biofilm of PEEK was less than that of Ti-6Al-4V, and that biofilm removal efficiency through treatment with photodynamic therapy on formed *S. mutans* and *A. actinomycetemcomitans* biofilms was more effective than with Ti-6Al-4V and Y-TZP [23]. PEEK had less reaction to bacteria and comparable biofilm removal ability via a non-antibiotic drug [26,43]; thus, replacing Ti-6Al-4V and Y-TZP with PEEK and PEKK might help alleviate inflammation.

The lower the ratio of ether to ketone in the molecular backbone of the PAEK, the more rigid the polymer chain [5], which means that PEKK has higher mechanical strength than PEEK [1], but from the perspective of dental science, the slightly higher strength of PEKK is a demerit. PEEK has a higher melting temperature and glass transition temperature than PEEK, which indicates that PEEK would be easier to process than PEKK. For PEKK, there is only one product form of disc. However, for PEEK, there are many product forms, such as single blocks (one-unit for crown or multi-unit for bridge) or discs (for large units), which could be suitable for either chair-side or in-lab dental CAD/CAM systems. Additionally, PEEK could be made into granules with a particle size distribution of 500 to 2500 μm or fine powder with an average particle size of 5 to 100 μm, which could be applied with additive manufacturing, compression molding, flame, or electrostatic spraying. The above advantages reveal that PEEK has developmental superiority and potential for use in current clinical digital dentistry.

Upon comprehensive consideration of the results of the present in vitro study, it is possible to assert that PAEKs, especially PEEK, represent suitable alternative materials to titanium alloy or zirconia implant–abutment. PEEK has compatible cytotoxicity and pro-inflammatory effects to titanium alloy and zirconia and is conducive to HOF adhesion. However, after implant–abutment insertion, many factors influence long-term success, such as cell interaction with materials, biofilm formation around abutments, and hygroscopicity or stability of materials in an aqueous environment. The current study only discusses human oral fibroblasts; however, other oral cells and fibroblasts need to be considered in the future. In future research, the biological safety of PEEK and stem cells, e.g., iPS, needs to be evaluated; meanwhile, in-depth studies on osseointegration and in vivo experiments need to be conducted to confirm the applicability of PEEK in dental implantology.

## 5. Conclusions

The present in vitro assessment emphasizes the importance of cell interaction with different abutment materials. Polyetheretherketone (PEEK) shows comparable cytotoxicity and pro-inflammatory effects to conventional implant–abutment materials (i.e., titanium alloy and zirconia). Additionally, PEEK is more conducive to the cell adhesion of human oral fibroblasts (HOF) than another polyaryletherketone family material, polyetherketoneketone (PEKK), in all aspects, which implies that PEEK has a better epithelial biological response. Therefore, it can be suggested that, from the perspective of biological behavior, PEEK is a suitable material as an implant–abutment.

## Figures and Tables

**Table 1:** Material assessed in the present study.

Trade Name (abbr.)	Main Composition	Manufacturer	Lot Number
**Metallic material**			
Coil (Ti-6Al-4V)	Titanium, aluminum, vanadium	S-Tech Corp. Tainan City, Taiwan	SM00940AF
**Ceramics material**			
90X10-HT (Y-TZP)	Zirconium dioxide, yttrium oxide	Aidite Technology Co., Ltd., Qin Huang Dao, Mainland China	W200614NG-1R
**Polymeric material**			
VESTAKEEP (PEEK)	polyetheretherketone	Evonik Japan Co., Tokyo, Japan	57781699
Pekkton ivory (PEKK)	polyetherketoneketone	Cendres+Métaux SA, Biel/Bienne, Switzerland	378526

**Table 2:** Primer sequences used for pro-inflammatory gene expression.

Gene	Primer Sequence
*GAPDH*	Forward primer: TGGTATCGTGGAAGGACTCATGA Reverse primer: ATGCCAGTGAGCTTCCCGTTCAG
*IL-1β*	Forward primer: CCACAGACCTTCCAGGAGAATG Reverse primer: GTGCAGTTCAGTGATCGTACAGG
*IL-6*	Forward primer: ACTCACCTCTTCAGAACGAATTG Reverse primer: CCATCTTTGGAAGGTTCAGGTTG
*TNF-α*	Forward primer: CTCTTCTGCCTGCTGCACTTTG Reverse primer: ATGGGCTACAGGCTTGTCACTC

GAPDH: glyceraldehyde 3-phosphate dehydrogenase; IL: interleukin; TNF-α: tumor necrosis factor-α.

**Table 3:** Surface characterization testing values for each abutment material (n = 5).

Materials	Contact Angle (Degree)	Surface Energy (mN/m)
Ti-6Al-4V	65.83 ± 3.28 ^a^	36.25 ± 4.09 ^A^
Y-TZP	76.92 ± 1.57 ^b^	27.38 ± 1.20 ^B^
PEEK	80.91 ± 1.63 ^c^	24.41 ± 1.19 ^C^
PEKK	84.03 ± 1.03 ^c^	22.19 ± 0.72 ^C^

All the values presented in the table were mean ± standard deviation (SD); within the same column, different letters indicate groups that are statistically different (*p* < 0.05).
